# Reductive carboxylation of glutamine as a potential target in acute myeloid leukemia

**DOI:** 10.18632/oncotarget.28474

**Published:** 2023-12-01

**Authors:** Alessia Roma, Lawrence D. Goodridge, Paul A. Spagnuolo

**Keywords:** acute myeloid leukemia, reductive carboxylation, metabolism, mitochondria, complex II

Acute myeloid leukemia (AML) is an aggressive cancer of the blood and bone marrow defined by poor patient outcomes and sub-optimal therapeutics [1]. Nonetheless, recent advancements in our understanding of AML biology bring optimism to improving patient outcomes for this devasting disease [2]. For example, the discovery and validation of metabolic vulnerabilities that are distinct to AML open new strategies for novel drug development. In fact, since 2017, a third of newly approved AML therapeutics have targeted metabolic abnormalities [2]. Thus, further identification and elucidation of metabolic vulnerabilities in AML could lead to novel therapies aimed at improving patient outcomes.

In Roma et. al., we reported that inhibiting complex II (CII) of the electron transport chain (ETC) targeted AML *in vivo* and *in vitro* without adversely affecting normal hematopoiesis [3]. CII inhibition resulted in a truncation of the tricarboxylic acid cycle (TCA) that significantly impaired synthesis of key metabolites (e.g., aspartate). Consequently, a reverse TCA cycle, referred to as glutamine-mediated reductive carboxylation, was activated to overcome this truncation [3]. As a result of activation, normal hematopoietic cells maintained anaplerosis while AML cells did not; failure to activate this pathway resulted in selective AML cell death. Similarly, inhibition of glutaminase further sensitized cells to CII inhibition, as glutamine was prevented from entering the TCA cycle [3]. These findings raise more questions worthy of future investigation. Specifically, does inhibiting CII offer any advantage over other ETC targets, can reductive carboxylation be directly targeted in AML, and are these targets (e.g., CII or reductive carboxylation) specific to an AML subtype? This editorial perspective presents an overview of recent findings regarding ETC impairment in AML and offers further insights into the topic.

Although strategies to inhibit the ETC have been explored, few examine direct CII inhibition. This is noteworthy, as CII possesses unique attributes. For example, CII does not contribute to the ETC proton gradient; therefore, its inhibition does not cause global ETC dysfunction [4]. Genetic mutations of complex I (CI) subunits cause severe and fatal conditions such as Leigh Syndrome (i.e., a fatal neurodegenerative disorder) whereas CII mutations typically cause benign neoplasms that occur later in life [5, 6]. The unfortunate early termination (due to neurotoxicity) of a clinical trialin AML patients using a novel CI inhibitor is further evidence of the difficulties in direct CI targeting [7]. No trial has yet tested direct CII inhibition in AML.

A second unique aspect of CII is its direct connection to the TCA cycle through succinate dehydrogenase [8]. Given this link, CII inhibition leads to a distinctive TCA cycle truncation and a dependence on reductive carboxylation [3, 9]. This metabolic pathway, in which glutamine is reduced to α-ketoglutarate, is often overlooked, yet it can sustain the synthesis of nucleotides and fatty acids when mitochondrial function is impaired [10, 11]. Studies in solid tumors use isotope tracing to show the compensatory effects of reductive carboxylation. The process is mainly mediated by isocitrate dehydrogenase, but also involves regulation of genes involved in the malate-aspartate shuttle and anaplerosis [10, 12–14]. The ability of cells to utilize reductive carboxylation may depend on the regulation of this genetic program and investigating differences in expression between malignant and non-malignant cells could reveal potential therapeutic targets [14, 15]. In a recent study, tumor growth in VHL-mutant clear cell renal cell carcinoma was impacted by the knockout of isocitrate dehydrogenase (IDH) 1 and IDH2, and showed a fundamental dependence on reductive carboxylation [15]. Pharmacologically, tumor growth was inhibited using an inhibitor of amidotransferases, which are enzymes that regulate reductive carboxylation [15]. Notably, VHL-mutant dependence on reductive carboxylation is a result of sustained activation of hypoxia inducible factor (HIF), a phenomenon that is also triggered by CII inhibition [16].

Targeting reductive carboxylation in AML may also be a promising avenue of future research. IDH 1/2 mutations, which occur in 20% of AML cases, impact activation of reductive carboxylation in other cancer types [17, 18]. If a similar trend is found in AML, patients with IDH mutations may be more responsive to drugs that elicit a shift towards this metabolic pathway. This hypothesis is supported by recent studies showing increased effectiveness of venetoclax, a BCL-2 inhibitor, in AML patients with IDH mutations [19, 20]. Venetoclax also imparts secondary actions of CI/CII inhibition and activation of reductive carboxylation [21–23]. Thus, venetoclax may further exploit a unique vulnerability in AML patients with IDH mutations via reductive carboxylation; however, this remains to be directly tested.

Additional areas of interest include assessing venetoclax in combination with inhibitors of glutaminase or IDH mutations. Experimentally, the glutaminase inhibitor CB-839 sensitized AML cells to drug-induced mitochondrial dysfunction by preventing glutamine entry into the TCA cycle and, thus, interfering with reductive carboxylation [3, 15]. Though not yet investigated clinically, synergy was observed between venetoclax and CB-839 in AML cells [24]. In AML patients with IDH mutations, early positive results from a phase I/II trial of venetoclax in combination enasidinib (i.e., targets IDH2 mutations) are reported with the combination providing a 55% overall response rate [25].

Identification and validation of novel and targetable metabolic weaknesses in AML is ongoing. The recent success of venetoclax and enasidinib, which directly or indirectly effect cell metabolism provides additional support for this approach. However, the central role of metabolism in physiological processes brings significant challenges in drug development, as evidenced by the recent failure of a clinical trial involving a CI inhibitor. One approach is to weaken tumor cell survival mechanisms. In this regard, exploring reductive carboxylation as a possible drug target could provide new avenues for optimizing existing treatments aimed at improving AML patient outcomes.
